# Quality of Life, Pulmonary Spirometry, and Dosage of Steroid in Asthmatic Patients with Polyposis after Endoscopic Sinus Surgery

**Published:** 2013

**Authors:** Bijan Khademi, Behrooz Gandomi, Mehdi Tarzi, Firoozeh Yeganeh

**Affiliations:** 1 *Department of Otorhinolaryngology, Khalili Hospital, Shiraz Institute for Cancer Research, Shiraz University of Medical Sciences, Shiraz, Iran.*

**Keywords:** Asthma, Endoscopy, FEV1, Polyp, Quality of Life

## Abstract

**Introduction:**

The association between asthma and sinonasal disease has been established for years. As sinonasal disease is one of the factors that exacerbates asthma, effective treatment of this disorder may also improve and stabilize the asthmatic condition. This study examines the outcome of endoscopic sinus surgery (ESS) on asthmatic patients with massive nasal polyposis.

**Materials and Methods:**

Forty-five asthmatic patients were included in the study. All were operated on and analyzed in our department. A questionnaire (SNOT-20) investigating the subjective evaluation of asthma and sinonasal states was presented to the patients, while objective evaluations including nasal rhinoscopy, forced expiratory volume in 1 second (FEV1), and steroid use were conducted with 1–2 years of follow up.

**Results:**

Quality of life (QoL) improved in 97.8% of patients, while clinical symptoms, emotional signs, and social signs improved in 97.7%, 84.4%, and 93.3%, respectively. Medication use for asthma showed a similar improvement, with approximately 80% of patients reducing and 75.6% of patients discontinuing steroid use. A total of 91.1% of patients showed improvement in post-operative FEV1.

**Conclusion:**

ESS achieved a beneficial effect on sinonasal and asthma symptomatology in patients with nasal polyps and asthma. QoL was also improved in these patients.

## Introduction

Asthma is a very common disorder around the world, and large numbers of patients with asthma associated with sinonasal disorders, particularly polyposis, are referred to clinics annually.

In general, 4% of the global population is affected by nasal polyposis of different causes (1).As this condition is an exacerbating factor for asthma, effective therapy of sinonasal polyposis may improve and stabilize the asthma epidemic (2).

Recently, endoscopic sinus surgery (ESS) has been regarded as one of the most effective surgical treatments in this regard. In the literature, a success rate of approximately 76% to 97.5% in primary surgery has been reported (3). Indeed, following ESS, a significant improvement in symptoms of asthmatic patients with polyposis has been reported (4).

In some studie‌‌s, the use of steroids following ESS has been reduced significantly. Furthermore, asthma and sinonasal polyposis can lead to individual and social dysfunction which may impact adversely quality of life (QoL) (5). 

We attempted to evaluate the effectiveness of ESS in asthmatic patients with sinonasal polyposis through spirometry (functional pulmonary‌ tests), monitoring required steroid dose, and QoL before and after surgery using the standard questionnaire SNOT-20.

## Materials and Methods

**Fig 1:**
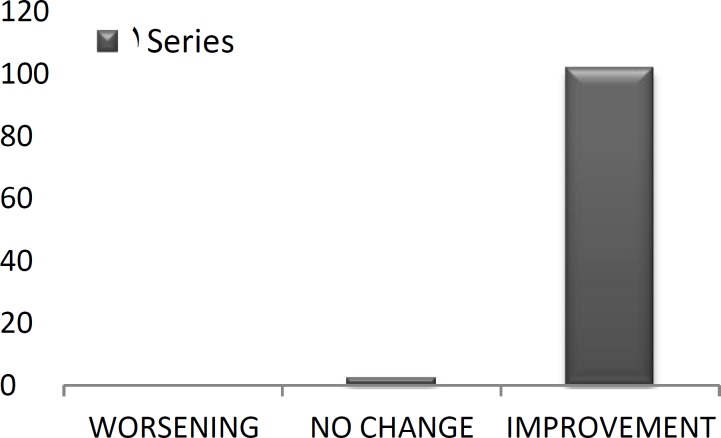
Change in clinical signs following ESS(%).

This study was performed in 45 asthmatic patients who were referred to the Mothari ear, nose, and throat (ENT) clinic associated with Shiraz Medical University from 2007–2008 due to sinonasal problems. Anterior rhinoscopy was performed and nasal polyposis was confirmed by a computed tomography (CT) scan of the paranasal sinuses. A standard questionnaire (SNOT-20; Figure 1) which includes questions relating to clinical, social, individual, and emotional problems was used in order to evaluate QoL. Questions in SNOT-20 can be classified in to three categories; the first 10 questions relate to clinical problems, the next seven relate to social and individual considerations, and the last three are concerned with emotional matters. The questionnaire was completed by an expert physician for all patients pre- and post-operatively. The severity of clinical problems scores ranges from (0): no problem, (1): mild problem; (2): moderate problem, to (3): severe problem. In addition, the dose of steroid used was recorded during the study.

In collaboration with a pulmonary specialist group, forced expiratory volume in 1 second (FEV1) was measured in all patients pre- and post-operatively.‌ The‌ procedure was performed in all patients by the same surgeon, and patients were followed up for 1–2 years post-operatively. Exclusion criteria included recent change in job, living area, or other exacerbating or mitigating factors unrelated to the procedure such as an increase or reduction in cigarette smoking, the presence of specific disorders, and new drug administration. 

## Results

Forty-five asthmatic patients pre- and post-ESS were included in this study. The mean age was 42.5 years (range, 23–62 years). Twenty-four patients (53.3%) were male, and 21 (46.7%) were female. Mean length of follow-up was 1.5 years (range, 1–2 years).

For confirmation of consistency in completing the SNOT-20 questionnaire, a Cranach’s α coefficient (variable >0.7) was applied, with an outcome of 0.87 before and 0.92 after the procedure.

A significant difference in QoL score before and after the procedure was observed (pair t-test: P<0.001). Before surgery, no significant difference between sex and QoL score was observed (P=0.6), while there was a significant difference after surgery (P=0.006). No significant differences between age and QoL score before (P=0.92) or after (P=0.24) ESS were detected.

Mean (±SD) FEV1 increased significantly following surgery (79.4 ± 4.7 vs. 90.1 ± 4.6; P<0.001). A strong correlation between QoL score and FEV1 before (P<0.001, R=−0.64) and after (P<0.001, R= −0.09) surgery was observed. A t-test statistical analysis showed 91.1% improvement in FEV1 following ESS.

**Fig 2:**
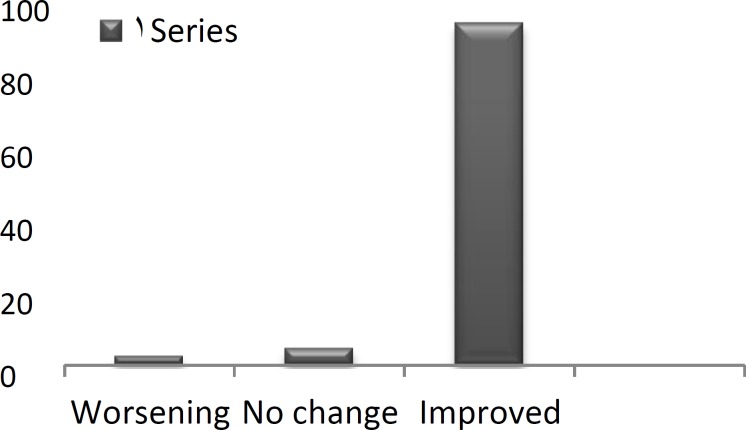
Change in social problems after ESS (%).

**Fig 3:**
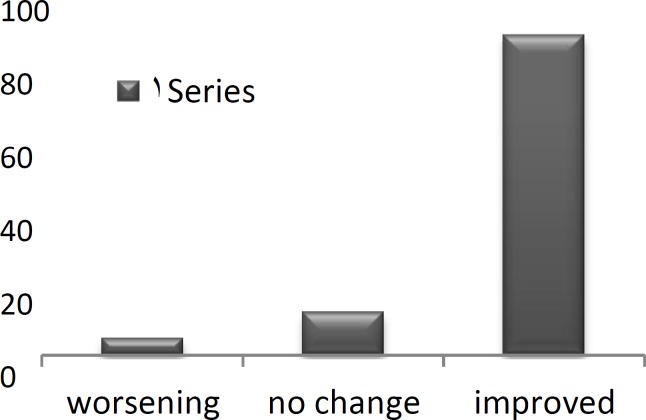
Change in emotional problems after ESS(%).

The vast majority of patients reported an improvement in Clinical problems (97.7%; Fig 1), social problems (93.3%; Fig 2), and emotional matters (84.4%; Fig 3) following surgery.

The use of steroids was also evaluated pre- and post-operatively. According to our survey, of 33 patients using oral steroids (5–10 mg prednisone), ‌27(81.8%) discontinued steroid used completely, two (6.1%) changed to inhaled steroids, and only four patients (12.1%) needed to continue oral steroids. Furthermore, of 12 patients using inhaled steroids before the procedure, seven (58.3%) discontinued steroid treatment completely, four (33.3%) continued with inhaled steroid treatment, and only one (8.3%) was required to use oral steroids after surgery. Overall, 34 patients (75.6%) discontinued steroid treatment after surgery.

## Discussion

Sinus disorders may intensify asthma for a number of reasons. First, post-nasal discharge stimulates lower respiratory airway in asthmatic patients (5); second, blocking of the β-adrenergic receptors related to bacterial toxins may contract the respiratory lumen (6); and finally, rhino-sino-bronchial reflex may result in lower respiratory tract spasm (6).

Recently, ESS has been shown to improve not only sinus problems, but also signs of asthma among patients (7,8). In this study, a strong correlation was observed between mean QoL score‌ in patients before and after surgery. Our statistical analysis indicates 97.8% improvement in QoL while Dursun et al previously reported 83% improvement in QoL outcome (9).

The influence of gender was not significant before surgery, but afterwards female patients fared less well than men. This was particularly the case for housewives, probably due to repeated exposure to washing materials and detergents, which stimulated asthma and caused a negative long-term impact on surgical results. 

No significant correlation between age and QoL of patients was observed before P = 0.12) or after (P= 0.24) surgery. In other words, ESS had similar odds of improving QoL among the older and younger age groups.

We found a significant improvement in various aspects of QoL following ESS. According to the survey, 97.7% improvement in clinical problems and 93.3% improvement in individual and social disturbances including sleep problems, daily and night fatigue and concentration were detected. Meanwhile, psycho-emotional problems such as restlessness, disappointment, sadness, and distraction resulting from asthma improved significantly after surgery (84.4%).

FEV1 is one of the most important indicators of pulmonary function, and has a direct relationship with severity of asthma. Mean baseline FEV1 (79.4 ± 4.7 %) increased following surgery (90.1 ± 4.6 %; P<0.001), reflecting the positive impact of ESS on asthma severity. Bart et al previously reported similar results, with a mean FEV1 of 70.6% following surgery (7).

After surgery, a remarkable correlation between FEV1 and QoL outcome in asthmatic patients was observed (P<0.001). With increasing FEV1 post operation, QoL scores decreased dramatically (R=−0.39), indicating improvement in QoL. Similar results in QoL outcome have also been reported previously (10-13).

Steroid therapy is a common treatment for asthma, but is associated with numerous side effects. In our study, ESS had a dramatic effect in reducing steroid usage. Of 33 patients using oral steroids, 27 (81.1%) discontinued steroid therapy, while seven of 12 patients (58.3%) using inhaled steroids were able to discontinue steroid treatment completely. Overall, 34 of 45 patients (75.6%) had no further need for steroid therapy following surgery, while an 80% reduction in steroid requirements was recorded postoperatively. Batra et al reported a comparable 70.6% decrease in steroid requirements, with 53% of patients discontinuing steroids after ESS (7). In a study by Dr. Dunlop et al, 20% of patients had reduced steroid inhaler requirements, with a significant reduction in oral-steroid usage (P<0.001) (12). 

## Conclusion

According to our study, ESS seems to be a very effective procedure to improve QoL outcomes among asthmatic patients and sino-nasal polyposis leading to a decline in the steroid requirements as a positive impact following surgery.
